# MR Imaging of Bone Marrow in Patients with Musculoskeletal Tumors

**DOI:** 10.1080/13577149977857

**Published:** 1999-03

**Authors:** David M. Panicek, Lawrence H. Schwartz

**Affiliations:** Department of Radiology Memorial Sloan-Kettering Cancer Center 1275 York Avenue NewYork NY 10021 USA

## Abstract

Knowledge of the appearances of normal bone marrow, metastases involving marrow, and therapy-related marrow changes shown by MR imaging is necessary in order to avoid misdiagnosis. This article reviews MR imaging techniques and the findings that allow distinction of normal yellow (fatty) marrow and red marrow from tumor in marrow, as well as the identification of marrow changes resulting from radiation therapy or treatment with marrow-stimulating drugs in patients with musculoskeletal tumors.


*Sarcoma* (1999) 3, 37–41

ISSUES IN IMAGING

### MR imaging of bone marrow in patients with musculoskeletal tumors

DAVID M. PANICEK & LAWRENCE H. SCHWARTZ

*Department of Radiology, Memorial Sloan-Kettering Cancer Center, 1275 York Avenue, New York, NY 10021, USA*

**Abstract**

Knowledge of the appearances of normal bone marrow, metastases involving marrow, and therapy-related marrow changes shown by MR imaging is necessary in order to avoid misdiagnosis. This article reviews MR imaging techniques and the findings that allow distinction of normal yellow (fatty) marrow and red marrow from tumor in marrow, as well as the identification of marrow changes resulting from radiation therapy or treatment with marrow-stimulating drugs in patients with musculoskeletal tumors.

**Key words**: *magnetic resonance (MR), bone marrow MR, bone marrow (effects of irradiation on), bone marrow neoplasms, tissue characterization.*

A quantity of bone marrow is visible on virtually every magnetic resonance (MR) imaging examination performed. Depending on factors such as patient age, the relative degree of hematopoietic activity present, and the body part being examined, various amounts of red marrow, yellow (fatty) marrow, or both will be seen on the images.[1–4] The MR imaging appearance of marrow is greatly affected by the specific MR pulse sequence employed, as well as by use of special options such as fat-suppression pulses. This article will review the common MR imaging techniques used for evaluating bone marrow and the MR imaging appearance of normal and abnormal marrow; of necessity, this is not a comprehensive review of MR imaging of the entire spectrum of marrow disorders.

### MR imaging techniques for evaluating marrow

On T1-weighted conventional spin-echo images, fatty marrow is readily identifiable by its high signal intensity, similar to that of subcutaneous fat, whereas red marrow shows intermediate to low signal intensity (Fig. 1a).[1] Regions of red marrow can be homogeneous or have a patchy distribution, and often show a feathery interface with adjacent fatty marrow—an important clue to their correct differentiation from tumor deposits. A small island of red marrow often can be characterized as such when one or more foci of central (bright) fat are visible within it on T1-weighted images—the so-called 'bull's-eye' sign, which has a reported sensitivity of 95% and a specificity of 99.5% for normal hematopoietic marrow.[5] In contrast, tumor deposits in marrow typically appear relatively homogeneous, and show low signal and no central high signal on T1-weighted images (Fig. 2).[1,5,6]

On T2-weighted conventional spin-echo images, fatty marrow has an intermediate signal intensity, similar to or slightly higher than that of red marrow (Fig. 1b), whereas tumor deposits manifest a moderately bright signal.[1,6] Tumor deposits in marrow often show a 'halo' sign on T2-weighted images, consisting of a rim of high signal surrounding a region of lower signal (Fig. 3); this sign has a reported sensitivity of 75% and a specificity of 99.5%.[5] Of note is the fact that fatty marrow (like fat in general) has moderately high signal on the newer 'fast' ('turbo') spin-echo T2-weighted sequences (Fig. 1c),[7] and thus can obscure adjacent tumor deposits or other pathologic processes. It is important to use frequency-selective fat-saturation (i.e., fat-suppression) pulses when evaluating marrow on T2-weighted fast spin-echo images (Figs 1d and 3) to improve the signal difference between tumor and normal marrow.[7,8] Another fat-suppression technique is the short-tau inversion recovery (STIR) sequence, which yields very high signal from abnormal marrow contrasted against very low signal from fat.[8,9]

Chemical shift images produced with in-phase and opposed-phase gradient echo pulse sequences are easily obtained on modern MR imaging units by appropriate selection of echo times (TE); for example,

Correspondence to: David M. Panicek, Department of Radiology, Memorial Sloan-Kettering Cancer Center, 1275 York Avenue, New York, NY 10021, USA. Tel: +1 (212) 639 5825; Fax: +1 (212) 794 4010; E-mail: panicekd@mskcc.org

1357-714X/99/010037-05 $9.00 © 1999 Taylor & Francis Ltd

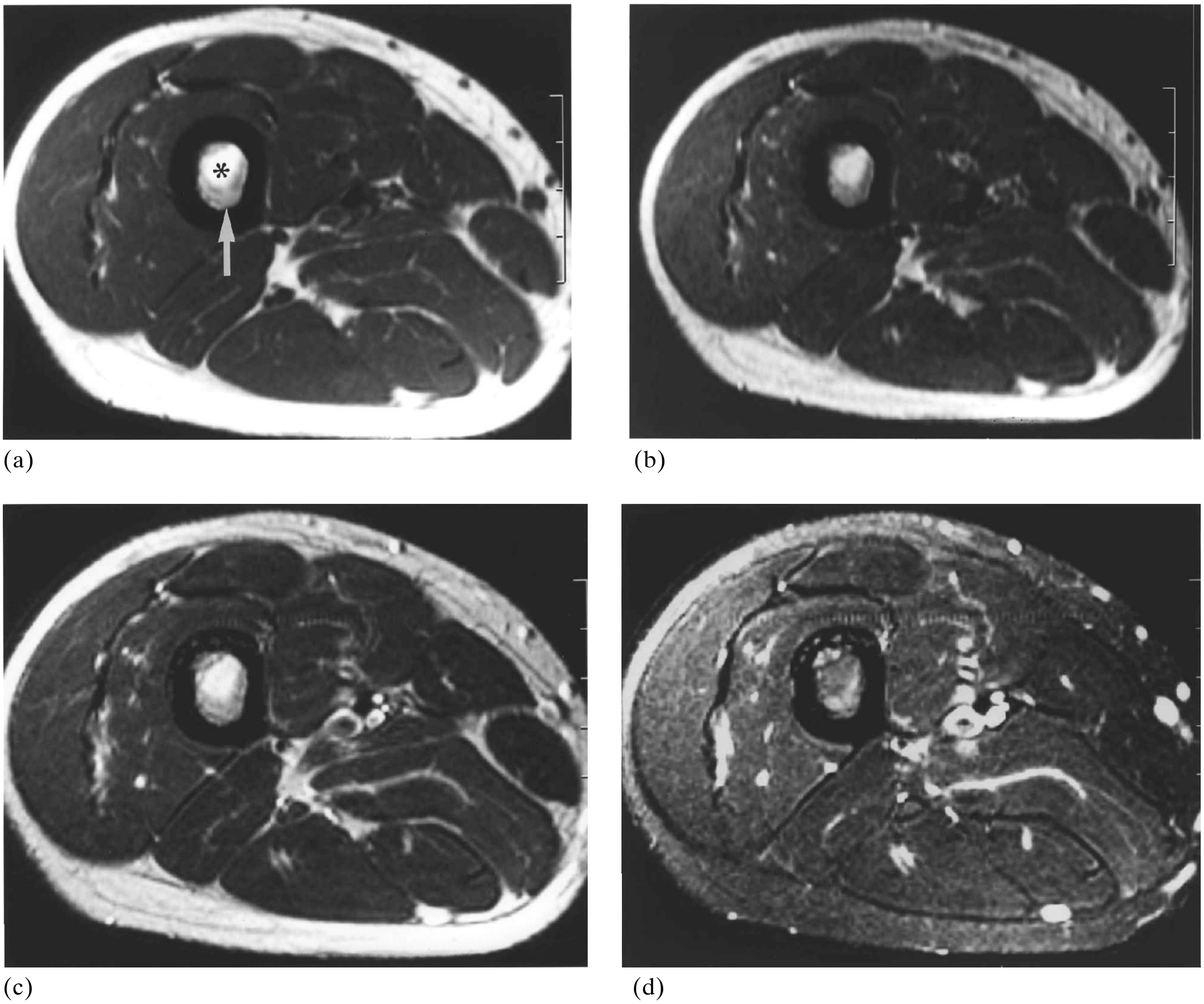

**Fig. 1.** *Normal red marrow and fatty marrow. T1-weighted spin-echo axial image (a) of normal marrow in femoral shaft shows subcortical distribution of red marrow (arrow), which has lower (grayer) signal than bright signal from more centrally located fatty marrow (asterisk). Similar signal intensity relationships are shown in marrow on T2-weighted conventional spin-echo (b) and T2-weighted fast spin-echo (c) axial images. T2-weighted fat-suppressed fast spin-echo axial image (d) at same anatomic level shows reversal of signal pattern compared to (a–c); relatively higher signal from subcortical red marrow could be confused with tumor (see Fig. 3).*

at 1.5 Tesla, images obtained with TE=2.1 msec are opposed-phase, and those obtained with TE=4.2 msec are in-phase. Because fatty marrow and red marrow both contain some amounts of both fat and water, the signal from either type of normal marrow will decrease to some extent on opposed-phase images (Fig. 4); in contrast, no fat is present within most primary or metastatic tumors within marrow, so tumor in marrow will not show such a signal loss.[10]

Administration of a gadolinium-based intravenous contrast material will cause most tumors in marrow to enhance, resulting in a higher signal on T1-weighted images (Fig. 5). Again, a fat-suppression technique should be used to increase the conspicuity of enhancing tumor against surrounding fatty marrow.

### Post-treatment changes

Within several weeks of radiation therapy, irradiated bone marrow shows increased signal on T1-weighted images, similar to that of subcutaneous fat, due to conversion to fatty marrow.[11,12] The interface between irradiated and nonirradiated marrow is typically straight and abrupt.

In patients in whom the bone marrow has been stimulated by anemia, regions of fatty marrow will reconvert to active red marrow, typically progressing from the central skeleton to more peripheral portions of the skeleton as the severity of anemia increases.[1] Reconversion to red marrow can be difficult to confidently distinguish from tumor involvement by MR imaging in some cases;[13] correlation with the expected pattern of metastasis for the specific tumor in a given patient can often help make this distinction.

Granulocyte-colony stimulating factor (G-CSF) is increasingly being administered to promote hematopoietic activity during chemotherapy in an attempt

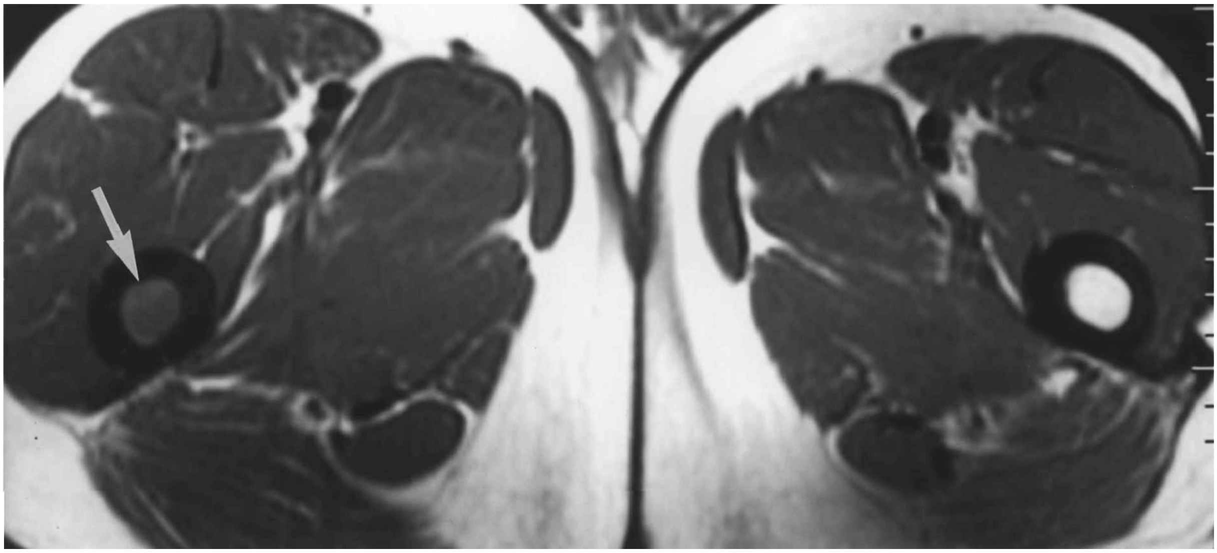

(a)

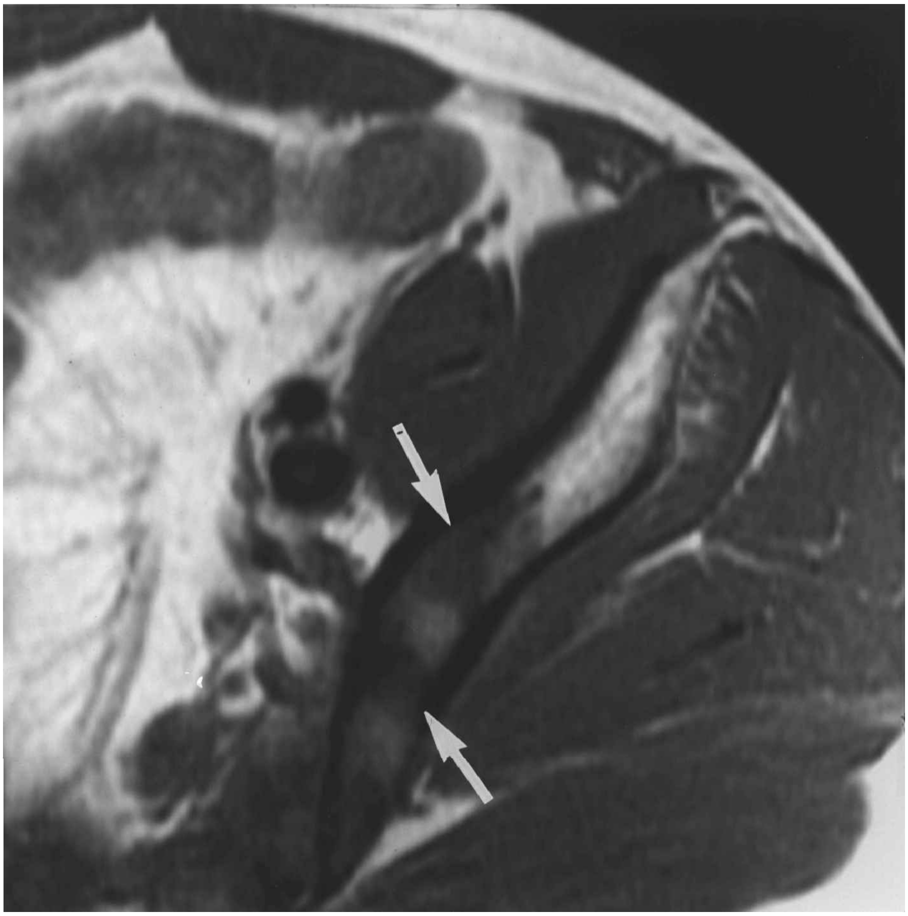

(b)

**Fig. 2.** *Metastatic tumor in marrow: T1-weighted imaging. T1-weighted spin-echo axial images of proximal femoral shafts (a) and left iliac wing (b) show relatively low signal from metastases (arrows) in marrow from liposarcoma (previously resected from soft tissues of left hip).*

to decrease the serious consequences of chemotherapy-induced neutropenia. This hematopoietic growth factor (and others like it) often causes multiple regions of red marrow to develop in previously fatty marrow, manifesting as regions of high signal on T2-weighted images (Fig. 6).[13,14] Knowledge of this common effect of G-CSF, combined with information about the individual patient's clinical status, usually makes it clear that the interval development of multiple new marrow metastases is unlikely in that patient.

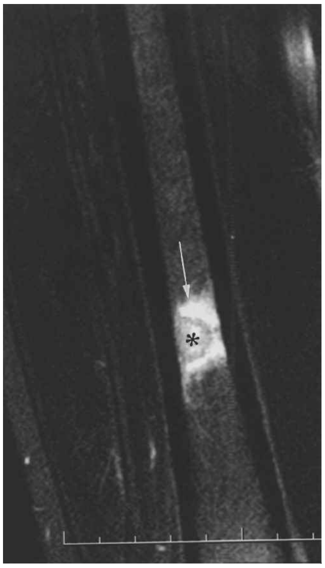

**Fig. 3.** *Metastatic tumor in marrow: halo sign. T2-weighted fat-suppressed fast spin-echo coronal image of femoral shaft containing marrow metastasis from lung carcinoma shows 'halo' sign, consisting of a rim of high signal (arrow) surrounding the lower signal tumor deposit (asterisk).*

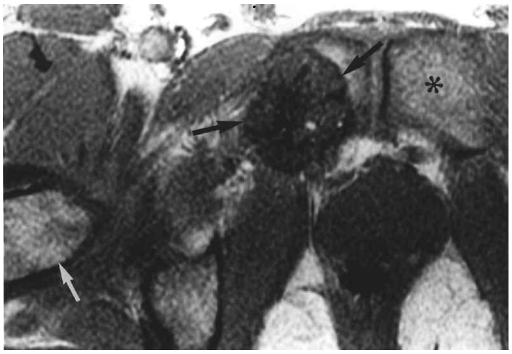

(a)

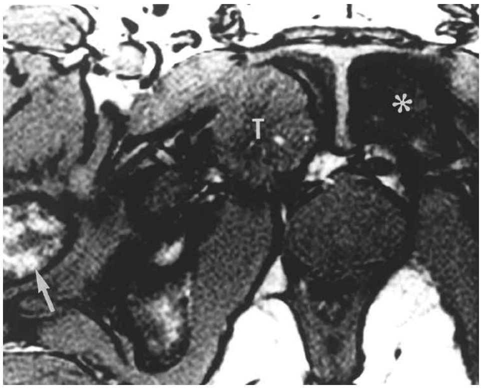

(b)

**Fig. 4.** *Metastatic tumor in marrow: chemical shift imaging. In-phase gradient echo axial image (a) of right lower hemipelvis shows deposit of metastatic renal cell carcinoma (black arrows) in marrow of right pubic bone. The tumor deposit has relatively low signal; fatty marrow (white arrow) has bright signal; and red marrow has intermediate signal (asterisk). On the corresponding opposed-phase image (b), the lesion (T) is much more conspicuous, as red marrow (asterisk) has become much darker due to cancellation of virtually all bright signal from the fat present within the red marrow. Areas of very fatty marrow (arrow) did not become as dark, because only a minimal amount of water was present to cancel only some of the bright signal from fat.*

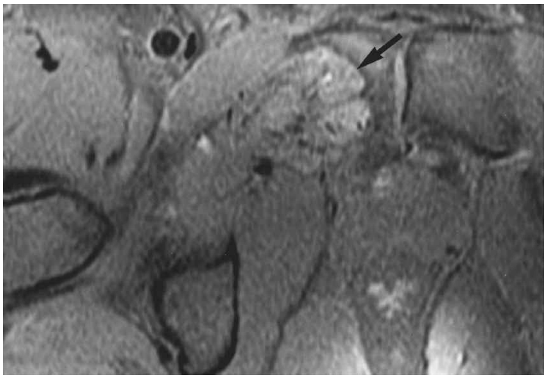

(a)

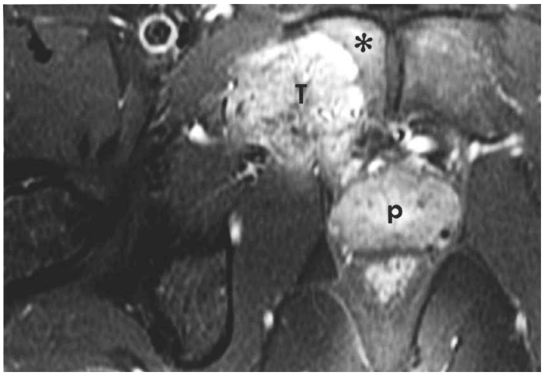

(b)

**Fig. 5.** *Metastatic tumor in marrow: gadolinium-enhanced imaging. Pre-injection fat-suppressed T1-weighted axial image (a) shows metastasis of renal carcinoma (arrow) in marrow of right pubic bone (same patient as in Fig. 4), with minimally higher signal than surrounding tissues. Following intravenous administration of gadolinium-based contrast material (b), the tumor deposit (T) markedly enhances, increasing its conspicuity. Note similar enhancement of prostate (p), minimal enhancement of edema in marrow medial to lesion (asterisk), and lack of enhancement in bone marrow elsewhere.*

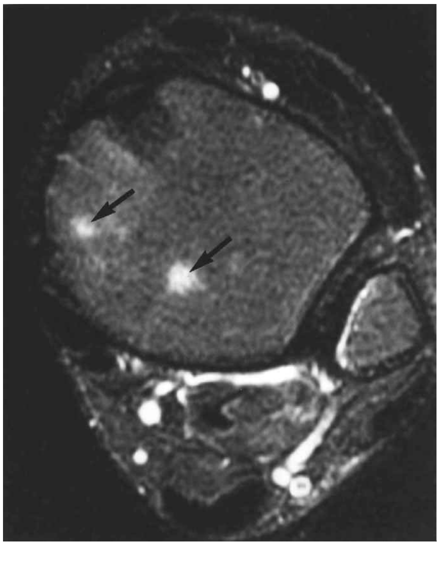

**Fig. 6.** *Growth factor effect. T2-weighted fat-suppressed fast spin-echo axial image of distal tibia in an adolescent patient who had recently completed preoperative chemotherapy, including G-CSF, for osteogenic sarcoma of femur. Multiple small foci of high signal intensity (arrows), representing islands of red marrow, developed in areas of previously fatty marrow as a result of therapy; these foci should not be mistaken for multiple new metastases.*

### References

1 Vogler III JB, Murphy WA. Bone marrow imaging. *Radiology* 1988; 168:679–93.

2 Ricci C, Cova M, Kang YS, *et al*. Normal age-related patterns of cellular and fatty bone marrow distribution in the axial skeleton: MR imaging study. *Radiology* 1990; 177:83–8.

3 Moore SG, Dawson KL. Red and yellow marrow in the femur: age-related changes in appearance at MR imaging. *Radiology* 1990; 175:219–23.

4 Taccone A, Oddone M, Dell'Acqua A, Occhi M, Ciccone MA. MRI 'road-map' of normal age-related bone marrow. II. Thorax, pelvis and extremities. *Pediatr Radiol* 1995; 25:596–606.

5 Schweitzer ME, Levine C, Mitchell DG, Gannon FH, Gomella LG. Bull's-eyes and halos: useful MR discriminators of osseous metastases. *Radiology* 1993; 188:249–52.

6 Ruzal-Shapiro C, Berdon WE, Cohen MD, Abramson SJ. MR imaging of diffuse bone marrow replacement in pediatric patients with cancer. *Radiology* 1991; 181:587–9.

7 Mirowitz SA. Fast scanning and fat-suppression MR imaging of musculoskeletal disorders. *Am J Roentgenol* 1993; 161:1147–57.

8 Shuman WP, Patten RM, Baron RL, Liddell RM, Conrad EU, Richardson ML. Comparison of STIR and spin-echo MR imaging at 1.5 T in 45 suspected extremity tumors: lesion conspicuity and extent. *Radiology* 1991; 179:247–52.

9 Pui MH, Chang SK. Comparison of inversion recovery fast spin-echo (FSE) with T2-weighted fat-saturated FSE and T1-weighted MR imaging in bone marrow lesion detection. *Skeletal Radiol* 1996; 25:149–52.

10 Disler DG, McCauley TR, Ratner LM, Kesack CD, Cooper JA. In-phase and out-of-phase MR imaging of bone marrow: prediction of neoplasia based on the detection of coexistent fat and water. *Am J Roentgenol* 1997; 169:1439–47.

11 Ramsey RG, Zacharias CE. MR imaging of the spine after radiation therapy: easily recognizable effects. *Am J Roentgenol* 1985; 144:1131–5.

12 Bloomlie V, Rofstad EK, Skjonsberg A, Tvera K, Lien HH. Female pelvic bone marrow: serial MR imaging before, during, and after radiation therapy. *Radiology* 1995; 194:537–43.

13 Fletcher BD, Wall JE, Hanna SL. Effect of hematopoietic growth factors on MR images of bone marrow in children undergoing chemotherapy. *Radiology* 1993; 189:745–51.

14 Vanel D, Missenard G, Le Cesne A, Guinebretiere JM. Red marrow recolonization induced by growth factors mimicking an increase in tumor volume during preoperative chemotherapy: MR study. *J Computer Assisted Tomography* 1997; 21:529–31.

## References

[PDF_00243] Blomlie V., Rofstad E. K., Skjønsberg A., Tverå K., Lien H. H. (1995). Female pelvic bone marrow: serial MR imaging before, during, and after radiation therapy.. Radiology.

[PDF_00235] Disler D. G., McCauley T. R., Ratner L. M., Kesack C. D., Cooper J. A. (1997). In-phase and out-of-phase MR imaging of bone marrow: prediction of neoplasia based on the detection of coexistent fat and water.. AJR Am J Roentgenol.

[PDF_00247] Fletcher B. D., Wall J. E., Hanna S. L. (1993). Effect of hematopoietic growth factors on MR images of bone marrow in children undergoing chemotherapy.. Radiology.

[PDF_00223] Mirowitz S. A. (1993). Fast scanning and fat-suppression MR imaging of musculoskeletal disorders.. AJR Am J Roentgenol.

[PDF_00208] Moore S. G., Dawson K. L. (1990). Red and yellow marrow in the femur: age-related changes in appearance at MR imaging.. Radiology.

[PDF_00231] Pui M. H., Chang S. K. (1996). Comparison of inversion recovery fast spin-echo (FSE) with T2-weighted fat-saturated FSE and T1-weighted MR imaging in bone marrow lesion detection.. Skeletal Radiol.

[PDF_00240] Ramsey R. G., Zacharias C. E. (1985). MR imaging of the spine after radiation therapy: easily recognizable effects.. AJR Am J Roentgenol.

[PDF_00204] Ricci C., Cova M., Kang Y. S., Yang A., Rahmouni A., Scott W. W., Zerhouni E. A. (1990). Normal age-related patterns of cellular and fatty bone marrow distribution in the axial skeleton: MR imaging study.. Radiology.

[PDF_00219] Ruzal-Shapiro C., Berdon W. E., Cohen M. D., Abramson S. J. (1991). MR imaging of diffuse bone marrow replacement in pediatric patients with cancer.. Radiology.

[PDF_00215] Schweitzer M. E., Levine C., Mitchell D. G., Gannon F. H., Gomella L. G. (1993). Bull's-eyes and halos: useful MR discriminators of osseous metastases.. Radiology.

[PDF_00226] Shuman W. P., Patten R. M., Baron R. L., Liddell R. M., Conrad E. U., Richardson M. L. (1991). Comparison of STIR and spin-echo MR imaging at 1.5 T in 45 suspected extremity tumors: lesion conspicuity and extent.. Radiology.

[PDF_00211] Taccone A., Oddone M., Dell'Acqua A. D., Occhi M., Ciccone M. A. (1995). MRI "road-map" of normal age-related bone marrow. II. Thorax, pelvis and extremities.. Pediatr Radiol.

[PDF_00251] Vanel D., Missenard G., Le Cesne A., Guinebretière J. M. (1997). Red marrow recolonization induced by growth factors mimicking an increase in tumor volume during preoperative chemotherapy: MR study.. J Comput Assist Tomogr.

[PDF_00202] Vogler J. B., Murphy W. A. (1988). Bone marrow imaging.. Radiology.

